# Lysosome biology in autophagy

**DOI:** 10.1038/s41421-020-0141-7

**Published:** 2020-02-11

**Authors:** Willa Wen-You Yim, Noboru Mizushima

**Affiliations:** Department of Biochemistry and Molecular Biology, Graduate School and Faculty of Medicine, The University of Tokyo, Tokyo, 113-0033 Japan

**Keywords:** Autophagy, Lysosomes

## Abstract

Autophagy is a major intracellular degradation system that derives its degradative abilities from the lysosome. The most well-studied form of autophagy is macroautophagy, which delivers cytoplasmic material to lysosomes via the double-membraned autophagosome. Other forms of autophagy, namely chaperone-mediated autophagy and microautophagy, occur directly on the lysosome. Besides providing the means for degradation, lysosomes are also involved in autophagy regulation and can become substrates of autophagy when damaged. During autophagy, they exhibit notable changes, including increased acidification, enhanced enzymatic activity, and perinuclear localization. Despite their importance to autophagy, details on autophagy-specific regulation of lysosomes remain relatively scarce. This review aims to provide a summary of current understanding on the behaviour of lysosomes during autophagy and outline unexplored areas of autophagy-specific lysosome research.

## Introduction

**Fig. 1:**
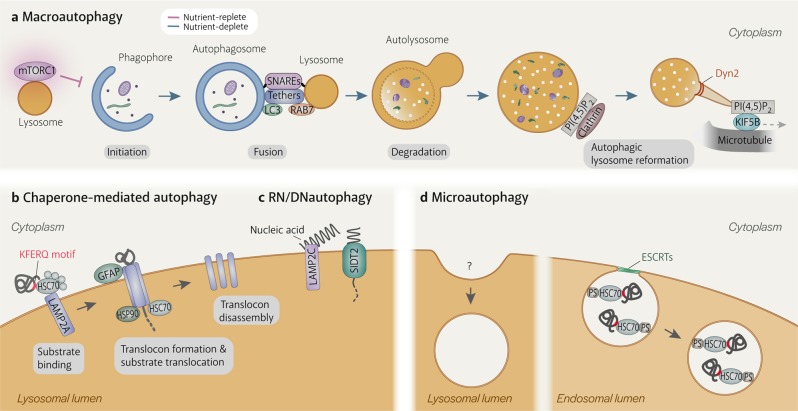
Autophagy processes. **a** Macroautophagy is the only autophagy process that involves another organelle, the autophagosome. It is induced when mTORC1 becomes inactivated upon dissociation from the lysosome. After the phagophore matures into a double-membraned autophagosome, the lysosome fuses with the outer autophagosomal membrane in a SNARE-dependent manner. Fusion is facilitated by tethering factors that bind to proteins on the autophagosome (e.g. LC3) and the lysosome (e.g. RAB7). Lysosomal enzymes degrade the inner autophagosomal membrane and sequestered material. Tubules extend from autolysosomes by KIF5B binding to clathrin-organised PI(4,5)P2 clusters on the autolysosomal membrane and moving away from the autolysosome on microtubules. The tubules are eventually cleaved from the autolysosome by Dyn2, generating new lysosomes. **b** Chaperone-mediated autophagy (CMA) involves the direct uptake of proteins with the KFERQ(-like) motif into lysosomes via a translocation complex consisting of LAMP2A monomers on the lysosomal membrane that is stabilised by GFAP and luminal HSP90. CMA substrates are delivered to LAMP2A by cytosolic HSC70 and other cytosolic chaperones. Substrate translocation is assisted by lysosomal HSC70. **c** RN/DNautophagy is the direct delivery of nucleic acids into lysosomes via the nucleic acid transporter, SIDT2. LAMP2C binds nucleic acids and potentially passes them to SIDT2 for translocation into the lysosomal lumen. **d** Microautophagy is the uptake of cytosolic material by invagination of the lysosomal membrane. Although it has been observed in lysosomes since the discovery of this organelle, mechanistic details are still scarce. Microautophagy in endosomes is more well-understood. Endosomal microautophagy substrates contain KFERQ(-like) motifs and are recognised by cytosolic HSC70 to be delivered to endosomes, where HSC70 binds to phosphatidylserine. Membrane deformation and eventually scission of intralumenal vesicle from the endosomal membrane are executed by the ESCRT machinery.

Autophagy refers to a set of pathways by which cytoplasmic material is delivered into the lysosome for degradation (Fig. 1). Starvation and other threats to cellular homeostasis strongly induce autophagy to acquire nutrients by recycling non-essential material or to eliminate harmful material. It comes mainly in three forms: macroautophagy, chaperone-mediated autophagy (CMA), and microautophagy^1^. Central to all of them is the lysosome, the characteristically acidic organelle with over 60 luminal hydrolases and important cellular regulators^2^.

While CMA and microautophagy take place directly on lysosomes (the former using a transmembrane protein translocation complex and the latter by membrane invagination), macroautophagy involves an additional organelle: the double-membraned autophagosome (Fig. 1a). Macroautophagy begins with the expansion of a piece of membrane, termed the phagophore, around cytoplasmic material that is targeted randomly or selectively with autophagy receptors. The expanding phagophore eventually resembles a sphere with a single opening, the sealing of which results in the autophagosome. Lysosomes fuse with the outer autophagosomal membrane (OAM), supplying acidic hydrolases that degrade the inner autophagosomal membrane (IAM) and sequestered material. The size of the autophagosome (~0.5–2 µm)^3^ enables macroautophagy to degrade material too large for CMA and microautophagy, which are restricted by the single-protein limitation of the translocation complex and the size of the lysosome (~0.5 µm)^3^, respectively. Protein aggregates, the ER, mitochondria, damaged lysosomes and bacteria are just a few of the targets of macroautophagy^1^.

In addition to serving as a source of degradative ability, lysosome is also involved in autophagy regulation, primarily through its relationship with the master kinase complex, mTORC1^4^. The activity of mTORC1 directly reflects intracellular and extracellular nutrient levels. An abundance in nutrients or growth factor signalling prompts mTORC1 to localize onto lysosomes, where it becomes activated to initiate growth-promoting processes and suppress macroautophagy by inhibiting the autophagy initiation complex^4^ and the nuclear translocation of the transcription factor EB (TFEB), which governs the transcription levels of lysosomal and autophagy genes^5–7^. Conversely, starvation causes mTORC1 to dissociate from lysosomes, leading to the induction of macroautophagy^4^ and likely microautophagy^8,9^. mTORC1 does not stay inactivated; its reactivation is required to replenish the lysosomal pool during prolonged starvation^10^. Constant cross-talk between lysosomes and autophagy, in terms of fusion and regulation, underlies steady autophagic flux.

In this review, we aim to provide a summary of the changes that lysosomes undergo as essential agents of macroautophagy, CMA, microautophagy, and RN/DNautophagy. We also discuss how lysosomes end up as substrates of macroautophagy (lysophagy). Here, the term ‘lysosome’ refers to acidic organelles with degradative potential and a layer of glycosylation on the luminal side of its membrane. We focus mainly on findings from mammalian studies and discuss what is still missing from our understanding of autophagy-specific lysosome regulation.

## Macroautophagy: autophagosome–lysosome fusion

A crucial step in macroautophagy is the autophagosome acquiring degradative enzymes by fusing with the lysosome (Fig. 1a). The high energy barrier of membrane fusion is overcome by the formation of a complex consisting of SNARE (soluble N-ethylmaleimide-sensitive factor attachment protein receptor) proteins embedded on either of the two membranes^11^. Autophagosome-lysosome fusion is executed by either of two SNARE complexes: STX17-SNAP29-VAMP7/VAMP8^12,13^ or STX7-SNAP29-YKT6^4^. SNARE complex formation is facilitated by tethering factors that hold the two vesicles close (Fig. 1a). For autophagosome-lysosome fusion, the HOPS complex^14^, PLEKHM1^15^, and EPG5^16^ play such a role by simultaneously interacting with proteins on both the autophagosomal membrane and the lysosomal membrane. PLEKHM1 binds to the lysosomal small GTPases, Arl8b^GTP^ and RAB7^GTP^, while also binding to LC3 on the autophagosome^15^. Similarly, EPG5 binds to RAB7^GTP^ and LC3^16^. The HOPS complex has a more extensive reach, being able to interact with lysosomal Arl8b^GTP^^17^ and the autophagosomal Qa-SNARE STX17, either directly^14^ or via Pacer^18,19^.

STX17 was the first autophagosomal SNARE identified in mammals. It is precisely recruited to fully formed autophagosomes^12,20^, thereby avoiding potential complications that could arise from lysosomes fusing with phagophores (discussed later). The mechanism underlying STX17 recruitment and its timing is still unclear. At its C-terminus is a hairpin loop made from two transmembrane domains with glycine zipper motifs that allows STX17 to insert into the OAM^7,9^. The C-terminal region containing the transmembrane domains is sufficient for accurate autophagosomal targeting and hence may contain an amino acid sequence that can sense changes in the OAM during autophagosome formation^12^. Alternatively, the timing of STX17 recruitment may be enforced by other proteins. ULK1 when free from Ser-423 phosphorylation has been reported to recruit STX17 to autophagosomes, where STX17 then preferentially binds SNAP29, resulting in the dissociation of ULK1^21^. STX17 has also been reported to bind directly to the autophagosomal protein, LC3^22^. However, further analyses should be conducted to confirm whether the strict timing of STX17 recruitment can be established by these methods of recruitment. A highly effective inhibitor of STX17 recruitment that does not suppress autophagosome maturation has been reported^23^ but its mode of action is unknown.

While acute depletion of STX17 activity by siRNA treatment^12^ or drug inhibition^23^ suppresses autophagic flux, chronic deficiency of STX17 has little effect^24^. This finding led to the discovery of a second autophagosomal SNARE, YKT6^24^, whose activity can compensate for STX17 deficiency. In mammalian cells, R-SNARE YKT6 forms a complex with Qa-SNARE STX7, and Qbc-SNARE SNAP29 (14A homologue was identified in *Drosophila*, in which YKT6 can replace VAMP7 to form a complex with Syx17 (the *Drosophila* homologue of STX17) and SNAP29^25^. In yeast, YKT6 is the sole autophagosomal SNARE^26,27^. Unlike STX17, YKT6 does not have transmembrane domains and must be modified with palmitoyl and farnesyl to associate with membranes^24^. YKT6 is also recruited to mature autophagosomes^24^, but the mechanism of this temporal regulation remains unknown.

Besides recruitment, the SNAREs involved in autophagosome-lysosome fusion are also subjected to other means of regulation. SNAP29 modified with O-linked *N*-acetylglucosamine^28^ and STX17 phosphorylated on its N-terminal domain^13^ cannot be incorporated into the SNARE complex. STX17 may also be suppressed by the ubiquitin conjugation enzyme BRUCE as STX17-positive autophagosomes accumulate in BRUCE-deficient cells^29^. Since BRUCE interacts with both STX17 and SNAP29^29^, it might interfere with STX17-SNAP29 binding on the autophagosome. On the other hand, VAMP7 competes with its SNARE-deficient isoform, VAMP7B, for incorporation into the SNARE complex. VAMP7 is favoured when VAMP7B is bound to DIPK2A^30^. When formed, the STX17-SNAP29-VAMP7 bundle must be stabilised by EPG5^16^ and ATG14L^31^. The YKT6-containing SNARE complex is less well-studied. In addition to molecular and genetic studies, structural information on both autophagosome-lysosome SNARE complexes will provide invaluable insights into the regulation of autophagosome-lysosome fusion.

The efficiency of autophagosome-lysosome fusion is also sensitive to the types and levels of particular phosphatidylinositol (PI) phosphates in the autophagosomal and lysosomal membranes. So far shown to be important are the reduction of PI(3,5)P_2_, production of PI4P, and suppression of PI(4,5)P_2_ appearance on either or both membranes. PI(3,5)P_2_ competes with actin for binding to cortactin on lysosomes and thus prevents the formation of stable actin filaments, which is crucial for efficient fusion. INPP5E dephosphorylates PI(3,5)P_2_ to PI3P, which allows cortactin to bind to actin^32^. Nevertheless, INPP5E activity must be restrained as PI(3,5)P_2_ must be present to activate TRPML1, the primary Ca^2+^ channel in the lysosomal membrane^33^. Although not yet directly demonstrated to be required for autophagosome-lysosome fusion, TRPML1 activity on lysosomes is still important for fusion as it contributes to the perinuclear localization of lysosomes^34^ and general lysosomal homeostasis^33^. Concurrently, PI4P is already present or being generated on both autophagosomal and lysosomal membranes^35,36^. The exact function of PI4P on the autophagosomal membrane is unclear but is proposed to be required for the association of fusion-promoting factors^35^. This has been shown for the lysosomal membrane, where the deliberate conversion of PI4P to PI(4,5)P_2_ causes the dissociation of RAB7 and its associated fusion-promoting effectors, including PLEKHM1^36^. Furthermore, reduced PI4P levels on the lysosomal membrane leads to tubulation^37^, which would likely hinder fusion. Eventually, PI4P is converted to PI(4,5)P_2_ but this occurs strictly after fusion^38,39^ as its premature appearance releases fusion-promoting factors from the lysosomal membrane^36^ in addition to inhibiting TRPML1 activity^39,40^. The appearance of PI(4,5)P_2_ is one of the steps the autolysosome undergoes to regenerate lysosomes, a process called autophagic lysosome reformation (ALR; described later)^41^.

Lysosomes fusing with spherical but unclosed phagophores has been observed in cells with defective autophagosome closure resulting from a deficiency in ATG conjugation proteins^20^ or the ESCRT-III subunit CHMP2A^42,43^. Degradation of the IAM is considerably delayed in such cells^20^, which would cause autophagic flux to stall and futile depletion of the lysosomal pool. Moreover, leaving lysosomal enzymes in the intermembrane space of autolysosomes runs the risk of them damaging the membrane and leaking into the cytoplasm. The many layers of regulation set upon SNARE recruitment, SNARE complex formation, and lipid composition ensure that autophagosome-lysosome fusion occurs only when the time is right.

## Macroautophagy/autophagy: degradation of the inner autophagosomal membrane and autophagic substrates

Degradation within autolysosomes starts with disruption of the IAM (Fig. 1a). In the vacuole of budding yeast, Atg15 was identified as the enzyme responsible for degrading the IAM (i.e. the membrane of autophagic bodies in the vacuole)^44,45^. An in vitro study found Atg15 to be a phospholipase that prefers phosphatidylserine^46^. The unidentified mammalian IAM lipase(s) might function similarly. In both organisms, the outer membrane (vacuolar membrane in yeast and OAM in mammalian cells) is spared from degradation despite being exposed to the IAM-degrading enzyme(s). The mechanism enabling resistance is unknown. One hypothesis is that the inner leaflet of the OAM lacks the substrates for the lipase, which is the mechanism proposed for the yeast vacuolar membrane against Atg15 activity^46^. Another hypothesis is that the OAM inherits membrane-protecting properties from the lysosomal membrane after fusion. This is supported by the observation of LAMP1, a lysosomal membrane protein, being present in the IAM of phagophores in CHMP2A-depleted cells^42^. As aforementioned, the IAM of phagophores is not readily degraded even after exposure to lysosomal enzymes^20^. However, the mechanism of enzymatic resistance is likely more complex since the IAM of phagophores can eventually be degraded, which is speculated to occur following autophagosomal closure^20^. The act of separating the phagophore membrane into the IAM and OAM during autophagosomal closure might confer different properties to the membranes, including the ability to resist degradation.

Lysosomal enzymes gain access to autophagic substrates after IAM degradation (Fig. 1a). More than 60 lysosomal hydrolases^2^ work in unison to digest the sequestered material, ranging from nucleic acids to bacteria^1^. Most of these enzymes have acidic pH optima^47^, making their function reliant on efficient acidification of autolysosomes. Poor lysosomal acidification is often attributed as the cause of impaired autophagy in diseases that are not apparently related to autophagy proteins^48–50^. Re-acidifying lysosomes by treatment with acidic nanoparticles^48^, drugs^51^ or by mTOR inhibition^52^ has been shown to restore autophagic flux, highlighting the importance of optimal enzymatic function.

The fate of catabolites generated from the degradation of autophagic substrates is poorly understood. It is widely accepted that they are exported from the lysosomes through numerous transporters on the lysosomal membrane and reused by the cell^40^. The activity of most transporters varies according to membrane voltage or intralysosomal proton levels^40^, which would make them reliant on V-ATPase activity. This is suggested by the finding that V-ATPase inhibition resulted in the accumulation of non-essential amino acids from a study on lysosomal metabolomics^53^. However, the same study also showed that V-ATPase inhibition did not affect the efflux of most essential amino acids, which was instead found to be regulated by mTORC1 activity in an SLC38A9-dependent manner^53^. Hence, catabolite efflux from lysosomes may be subjected to several regulatory mechanisms that are not just based on lysosomal membrane properties. These mechanisms are still mostly unclear, especially with respect to lipid egress. NPC1, NPC2^54^ and LIMP2^55^ have been identified to transport cholesterol from the lysosomal lumen to the lysosomal membrane but little is known about the transport of other lipid products. As indicated by recent studies, lipids may be transferred from lysosomal membranes to other organellar membranes via membrane contact sites^56^. Lipid egress should be tightly regulated to prevent the lysosomal membrane from losing lipids essential to its function. Since the release of catabolites from lysosome is essential for the cell to adapt to starvation, further investigations should be conducted, particular for catabolites besides amino acids and cholesterol, to gain a complete understanding of this process.

## Macroautophagy: autophagic lysosome reformation

During prolonged macroautophagy, persistent autophagosome-lysosome fusion results in most, if not all, lysosomes being incorporated into autolysosomes^10^. Besides lysosomal biogenesis, the cell replenishes its lysosome stores by autophagic lysosome reformation (ALR), a process by which lysosomes are regenerated from autolysosomes during prolonged starvation and other lysosome-depleting circumstances^57,58^. Without ALR, the cell struggles to adapt to starvation and becomes more susceptible to cell death^57^.

ALR begins with the reactivation of mTORC1^10,57^, initiated by lysosomal calcium-based negative feedback^59^ as well as increased amino acid levels in the cytosol^60,61^ and the lysosomes^62^. The link between mTORC1 reactivation and ALR initiation is not known but may be the phosphorylation of UVRAG by reactivated mTOR. Phosphorylated UVRAG activates the class III PI 3-kinase VPS34 to generate PI3P on autolysosomes, whose levels may determine rate of tubulation^57^. PI3P is also implicated in the recruitment of spastizin and spatacsin, two proteins of unknown function but have been reported to be essential for autolysosomal tubule formation^63^. RAB7 must also be removed from autolysosomes before ALR can take place^10^. RAB7 enforces lysosomal association to dynein for perinuclear localization which facilitates autophagosome-lysosome fusion^64^. Post-fusion, autolysosomes might dispense with dynein and instead associate with kinesin, which drives autolysosomal tubulation.

Tubule formation requires the conversion of autolysosomal PI4P to PI(4,5)P_2_ by the PI4P 5-kinases, PIP5K1A and PIP5K1B. Clathrin binds PI(4,5)P_2_ via AP2 and organises PI(4,5)P_2_ into clusters on the autolysosomal membrane^41^. Tubules are generated by kinesin motor protein KIF5B^41,65^ binding to the PI(4,5)P_2_ clusters and presumably pulling on the autolysosomal membrane while moving away on microtubules^65^. Tubulation is facilitated by WHAMM-mediated actin formation at the autolysosome core and at the base of the tubules^66^. It is unclear what prevents the autolysosome core from moving with KIF5B; it may be held in place by actin^66^ or by a dynein-based counterforce as a balance between dynein-driven and kinesin-driven movement has been reported to be important for tubulation^34,67,68^. This balance is proposed to be maintained by the lysosomal Ca^2+^ channel TRPML1, which has also been implicated in scission of the tubules^34^.

During tubulation, the movement of lysosomal luminal contents is restricted to prevent them from entering the tubules and potentially disrupting the membrane^57^. This is achieved by an unidentified mechanism dependent on optimal levels of PI4P^37^ and PI3P^57^. The autolysosomal tubules are eventually severed by the GTPase Dynamin 2 (Dyn2) powered by hydrolysis of GTP^69^. In Dyn2-depleted cells, electron-dense tubules extending from autolysosomes were observed^69^, suggesting that lysosomal enzymes are only weakly retained in the autolysosomal core. The new lysosomes derived from the severed tubules eventually become acidic and capable of hydrolysis^10^, perhaps by transiently fusing with late endosomes or mature lysosomes^70^.

## Autophagy regulation by lysosomes

Starvation-induced inactivation of mTORC1 is one of the main inducers of autophagy (except perhaps for CMA). When the cell has sufficient levels of nutrients, mTORC1 is recruited to lysosomes by a complex composed of Rag-GTPases. The Rag complex is in turn tethered to the lysosomal membrane via another multi-subunit complex called Ragulator that interacts with the lysosomal V-ATPase and the amino acid transporter SLC38A9^71,72^. Both the Rag complex and Ragulator must be in their ‘active’ conformations^71,72^ and located in RAB7-free microdomains on the lysosomal membrane^73^ to recruit mTORC1. When on the lysosomal membrane, mTORC1 is activated by GTP-bound Rheb. Activated mTORC1 suppresses macroautophagy by phosphorylating ULK1 and ATG13 of the autophagy initiation complex, preventing its activation^71,72^.

mTORC1 activation is regulated by nutrient levels in the cytosol and the lysosome^71,72^. Cytosolic nutrient levels are detected by protein sensors that inform Rag and Ragulator conformations and in turn determine whether mTORC1 is recruited to lysosomes for activation^71,72^. A drop in nutrient levels turns off the mechanism to recruit mTORC1, resulting in mTORC1 inactivation. Autophagy is initiated and lysosomes begin receiving large numbers of macromolecules. Within lysosomes, some macromolecules may trigger signalling that promotes autophagic flux, such as mitochondrial DNA and its induction of TLR9 signalling^39^. The macromolecules are gradually broken into their constituents, such as amino acids that would be used in the synthesis of essential proteins. However, amino acid efflux during starvation requires mTORC1 reactivation^53^, which is achieved by the lysosomal V-ATPase strengthening Ragulator-Rag interaction in response to the rise in intralysosomal amino acid levels and enabling mTORC1’s lysosomal recruitment^62^. Amino acid efflux is amplified when free arginine in the cytosol and lysosomal lumen activates SLC38A9, an amino acid transporter and another positive regulator of mTORC1 activity^74,75^. mTORC1 reactivation also initiates ALR, replenishing the lysosomal pool (^10^; see previous section). Should intracellular nutrient levels remain low, mTORC1 will become inactivated again and the cycle will continue till starvation is resolved.

## Lysosomal activation during autophagy

Autophagic flux during starvation is supported by elevated lysosomal activity. Starvation-induced inactivation of mTORC1 removes its suppression on TFEB, which then translocates to the nucleus, where it upregulates the transcription of lysosomal and autophagy genes, supporting the production of lysosomes and autophagosomes^5–7^. Lysosomes associate with dynein instead of kinesin to move to the perinuclear region, where most autophagosome-lysosome fusion occurs^64^. Perinuclear lysosomes are more acidic^76–79^, which enhances enzymatic function^80^ to efficiently degrade autophagic substrates.

Although starvation-induced lysosomal activation is mainly attributed to mTORC1 inhibition, certain findings indicate that autophagy proteins may also be required. A study on the relationship between lysosomes and autophagy found that lysosomes in cells without ATG5 or ATG7 (members of the ATG conjugation system) failed to acidify and showed no enhancement in enzymatic activity in response to starvation or mTORC1 inhibition despite TFEB activity being unaffected^81^. Consistent with this is the finding that amino acid starvation-induced V-ATPase assembly is independent of mTORC1 activity^74^, suggesting that lysosomal activation, at least in terms of acidification, is not regulated by mTORC1 activity and may be linked to autophagosome formation. However, acidification of lysosomes in normal cells was not observed after initiating mTORC1-independent autophagy by trehalose treatment^81^. Furthermore, a separate study observed acidification of lysosomes in ATG5-deficient cells starved of amino acids and serum^77^. The effect of autophagosome formation on lysosomal function should be further investigated.

## Quality control of lysosomes by lysophagy

Despite being fortified with the glycocalyx, a 5–12 nm-thick layer of sugar residues on the luminal side of its membrane proteins^82^, the lysosomal membrane remains susceptible to damage by various stressors such as drug-mediated/disease-related lysosomotropism, the loss of stabilising proteins, and trapped infectious agents^83^. When the lysosomal membrane is breached, lysosomal function is lost. Moreover, lysosomal contents are released into the cytoplasm, resulting in damage to cytoplasmic components and, ultimately, cell death^83^.

**Fig. 2:**
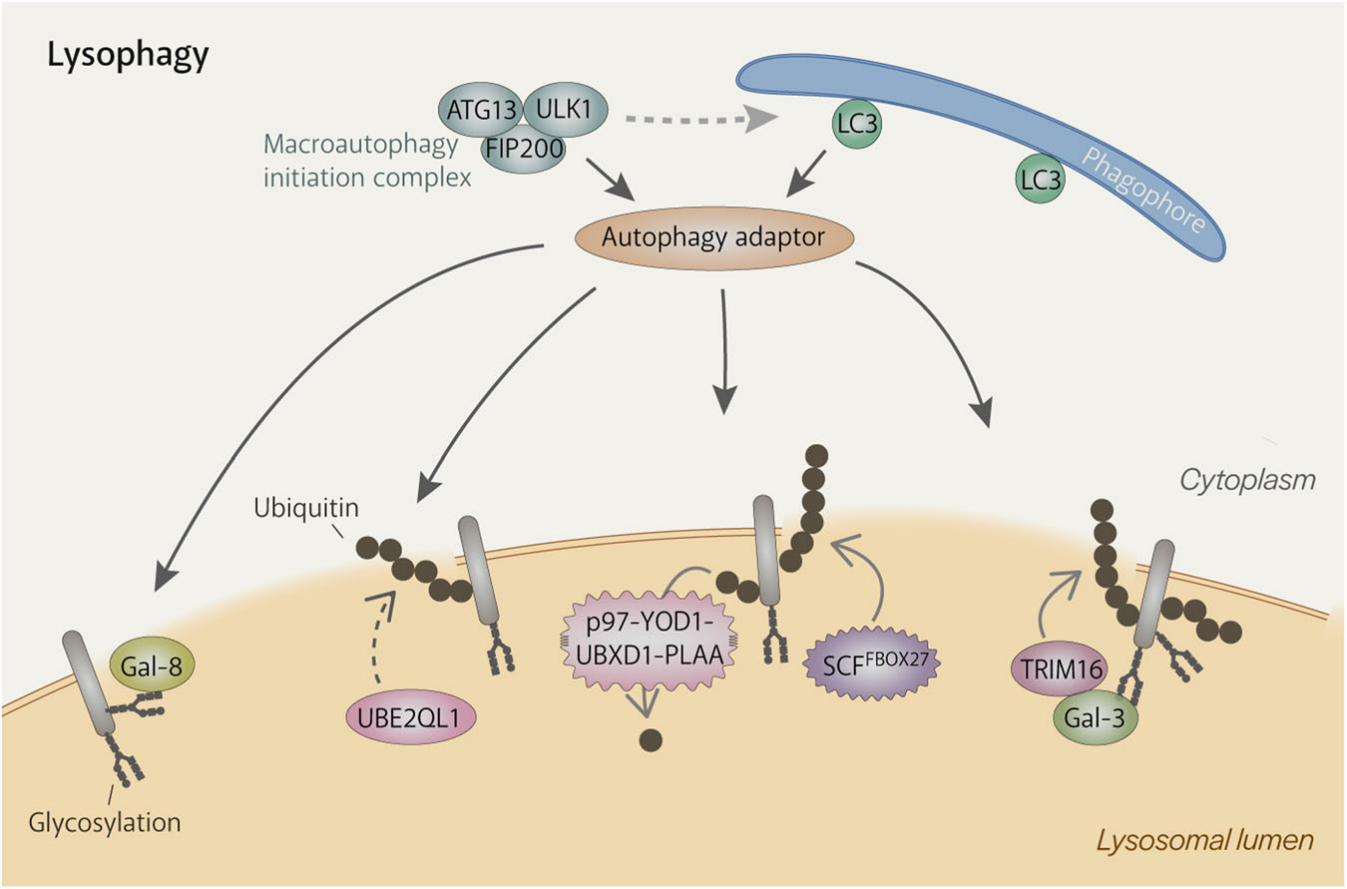
Mechanisms of autophagy machinery recruitment to damaged lysosomes. Extensive damage to the lysosomal membrane allows cytosolic proteins to pass through freely, including glycan-binding galectins and ubiquitin ligases. Damaged lysosomes are heavily ubiquitinated, which is carried out by ubiquitylation enzymes such as UBE2QL1 (an E2 enzyme), TRIM16 (an E3 ligase) and SCFFBOX27 (an E3 ligase). K48-linked ubiquitin chains are removed by the p97-YOD1-UBXD1-PLAA complex to emphasise the presence of K63-linked chains, which are preferred by the autophagy machinery. Autophagy adaptors bind either directly to galectins (e.g. NDP52) or to ubiquitin (e.g. p62, OPTN, TAX1BP1). They then recruit the autophagy machinery, including the initiation complex, and serve to promote the formation of the autophagosome specifically around the damaged lysosome.

Lysophagy is the engulfment of damaged lysosomes by autophagosomes with the aim of limiting the spread of damage (Fig. 2). It is employed when ESCRT-mediated repair, the first line of defence, proves to be insufficient^84,85^. Although direct evidence is still lacking, lysophagy most likely targets severely damaged lysosomes whose membranes no longer act as barriers against free movement of proteins and other material. This is indicated by observations of autophagy-promoting proteins and modifications on the luminal side of the lysosomal membrane and that such proteins (e.g. ubiquitin ligases^86–88^) appear on damaged lysosomes after ESCRT recruitment^84,85^.

The autophagy machinery is primarily recruited by ubiquitination of damaged lysosomes^89,90^. Membrane damage exposes the otherwise hidden glycocalyx, which recruits galectins (Gals). Amongst them is Gal-3, which draws the E3 ligase TRIM16 into the lumen of damaged lysosomes. TRIM16 then mediates ubiquitination of the damaged lysosomes (actual targets are still unidentified) and also recruits upstream autophagic factors, ULK1, Beclin1 and ATG16L1^91^. Another E3 ligase involved in lysophagy was identified as the SKP1-CUL1-F-box protein 27 (SCF^FBXO27^) ubiquitin ligase complex^88^, which can directly bind to the exposed glycocalyx and associate with the damaged membrane via myristoylated FBXO27. SCF^FBXO27^ ubiquitinates SNARE proteins and lysosomal membrane proteins^88^. Cells deficient in either E3 ligase experienced impaired ubiquitination and lysophagy^88,91^ but still had residual ubiquitination that may have been produced by the other E3 ligase or other unidentified E3 ligases. The latter is more likely as both E3 ligases were not found to function downstream of UBE2QL1, which was identified to be a lysophagy-mediating E2 ligase^86^. UBE2QL1 enters the lumen of damaged lysosomes by an unknown mechanism and mediates mostly K48-linked ubiquitination of the luminal ends of lysosomal membrane proteins^86^. UBE2QL1 activity is important for recruitment of the autophagy receptors, p62 and TAX1BP1, and p97^86^. The latter is part of a complex with YOD1, UBXD1 and PLAA, that removes K48-linked ubiquitin and thus emphasises the presence of K63-linked ubiquitin, which is preferred by the autophagy machinery^87,92^. The profile of the ubiquitination substrates on damaged lysosomes and the types of ubiquitin linkage utilised in lysophagy are still unclear.

Although the upstream autophagy factor ATG16L1 can directly recognise ubiquitin^90^, the autophagy machinery is mainly recruited by the binding of autophagy receptors to ubiquitin. p62^86–90^, NDP52^93^, TAX1BP1^86^ and OPTN^94^ are autophagy receptors that have ubiquitin-binding domains^95^ and known to localize to damaged lysosomes. Autophagy receptors bind autophagic substrates and LC3 on the phagophore at the same time, encouraging the phagophore to expand around autophagic substrates^95^. Recent work has shed new light on the role of autophagy receptors. NDP52 was discovered to be able to interact with subunits of the autophagy initiation complex, FIP200^96^ and ULK1^97^, and TANK-binding kinase 1 (TBK1)^96,97^ and thus specifically initialise autophagosome formation around damaged lysosomes. p62 can also recruit FIP200 to ubiquitin condensates and probably does the same during lysophagy^98^.

Lysophagy is supported by mTORC1 inactivation, the resulting TFEB activation, and AMPK activation, which are mediated by galectins^99^. Gal-8 interacts with the Ragulator-Rag signalling machinery to cause mTORC1 dissociation and subsequent inactivation while Gal-9 recruits TAK1, which activates AMPK by phosphorylation^99^. Activated AMPK then phosphorylates ULK1 and ATG13 of the autophagy initiation complex, enhancing autophagic activity and thus lysosome clearance^99^.

## Chaperone-mediated autophagy

Chaperone-mediated autophagy (CMA) is the direct translocation of protein substrates from the cytosol into the lysosomal lumen mediated by LAMP2A^100^, one of the three splice variants of the *LAMP2* gene^101^. The generation of mice with a liver-specific deficiency of LAMP2A revealed that CMA is important for liver metabolism^102^ and increased CMA activity has been observed in response to a variety of conditions such as starvation, hypoxia, and oxidative stress^103^.

CMA substrates are delivered to lysosomes by HSC70, a cytosolic chaperone. HSC70 binds to a five amino acid-long motif, KFERQ or a variation of which, on the CMA substrate and brings it to LAMP2A on the lysosomal membrane (Fig. 1b). Both HSC70 and the CMA substrate then associate with the cytosolic region of LAMP2A, triggering the formation of multimeric LAMP2A complex^104,105^. Exactly how multimerisation of LAMP2A, a single-pass transmembrane protein, results in a transmembrane pore has yet to be determined. Multimerisation can only occur in cholesterol-poor regions of the lysosomal membrane^106^ and the resulting complex has to be stabilised by another lysosomal membrane protein, GFAP^107^, and luminal HSP90^104^ before it can translocate CMA substrates. The translocation channel of the complex is only wide enough to accommodate proteins that have been unfolded by HSC70 and several other chaperones in the cytosol^108,109^. Translocation is assisted by HSC70 in the lysosomal lumen^110^. After the substrate reaches the lysosomal lumen, substrate-free cytosolic HSC70 on the lysosomal membrane surface disperses the LAMP2A complex^104^. Since LAMP2A is the defining factor of CMA^103^, a full characterization of this protein, including structural studies of full-length LAMP2A and the translocation complex, would provide a significant advancement to current understanding of CMA.

While it is generally accepted that the rate of CMA is regulated by the levels of LAMP2A and its multimerisation efficiency^103^, the signalling upstream remains mostly unclear. Unlike other autophagy processes, mTORC1 does not regulate CMA^111^. mTORC2, however, influences the rate of LAMP2A multimerisation by activating Akt, which then phosphorylates GFAP, preventing it from stabilising LAMP2A complexes^107^. During prolonged starvation, Akt is inactivated by the phosphatase PHLPP1, leading to higher levels of GFAP that can associate with LAMP2A complexes^112^. The phosphatase for GFAP, if there is one, has not been identified. As mTORC2 and Akt levels on CMA-active lysosomes during prolonged starvation stay relatively stable, translocation complex formation depends mainly on PHLPP1’s recruitment to the lysosome^112^. The signal for recruitment of PHLPP1 and how CMA is activated only after prolonged starvation are two of the many unanswered questions on the regulation of CMA.

## RN/DNautophagy

RN/DNautophagy (RDA) refers to the autophagic pathway by which nucleic acids are taken up directly by lysosomes for degradation (Fig. 1c). Its discovery began with the finding that LAMP2C was capable of binding RNA and DNA^113,114^. Subsequently, it was shown that isolated lysosomes could take up nucleic acids and that LAMP2-deficient lysosomes were less efficient in doing so^113,114^. Although LAMP2B can also bind nucleic acids^113,115^, its affinity for nucleic acids is much weaker than that of LAMP2C^113–115^. LAMP2C was thus named the first RDA receptor^113,114^.

The observation that LAMP2-deficient lysosomes had decreased but remaining RDA activity^113,114^ prompted the search for other RDA receptors. This led to the identification of SIDT2^116,117^, a putative double-stranded RNA transporter previously reported to localize to lysosomes^118^. SIDT2 is able to independently transport nucleic acids across the lysosomal membrane^116,117^ unlike LAMP2C, whose inability to multimerise renders it incapable of doing so^119^. Hence, SIDT2 is regarded to be the more important of the two^116^. LAMP2C can interact with SIDT2^116^, suggesting that it might pass its bound DNA or RNA to SIDT2 for delivery into lysosomes, but this has yet to be demonstrated. Furthermore, whether SIDT2 displays substrate selectivity is still unknown. By contrast, LAMP2C has been shown to prefer guanine-rich sequences^120^. Studies outside of the autophagy field have reported that SIDT2 exports viral RNA from lysosomes into the cytoplasm^121^ and that it has sodium ion transporter activity^122^. Whether these functions are related to RDA should be investigated.

The physiological relevance of RDA might involve the degradation of unwanted nucleic acids (e.g. viral DNA and mitochondrial DNA) as indicated by the increased mortality rates experienced by SIDT2-deficient mice post-viral infection^121^. However, this same study reported an accumulation of RNA in lysosomes and that the function of SIDT2 is to export RNA from lysosomes into the cytosol^121^. Additionally, several studies characterizing SIDT2-knockout mice have reported defects in insulin secretion^123,124^, hepatic lipid metabolism^125,126^ and autophagic flux^127^, which do not seem to involve nucleic acid degradation but should be investigated to clarify the physiological role of RDA.

## Microautophagy

Microautophagy refers to the process whereby lysosomes directly engulf cytosolic material by membrane invaginations (Fig. 1d). Although over 50 years have passed since it was first described^128^, little is known about the molecular machinery and regulation of microautophagy in mammals. This is mainly due to the difficulty in observing membrane invaginations in the small lysosomes of mammalian cells and also to the lack of robust assays to specifically measure the rate of microautophagy.

In contrast to the lysosome setup in mammalian cells, yeast cells typically have one large degradative ‘lysosome’, called the vacuole, whose size makes microautophagy easier to observe and studies with yeast cells have yielded several critical findings revealing the scope of microautophagy. Proteins and organelles were found to be targeted by vacuolar microautophagy and substrates differ according to the cell’s condition. Peroxisomes were found to be eliminated by microautophagy in yeast when methanol is replaced by glucose as an energy source^129,130^. Nutrient deprivation induces microautophagy of portions of the nucleus via nucleus-vacuole junctions^131^ and lipid droplets^132^. The ER is taken up during ER stress^133^. Studies with yeast have also determined that microautophagy is mediated by the ESCRT machinery^134^ and regulated by TORC1 activity^135^. The importance of GTP availability, membrane fluidity and membrane potential was also discovered, pointing to the existence of unidentified factors^136^.

On the mammalian front, microautophagy was recently discovered to occur on endosomes^137^. Termed endosomal microautophagy (eMI), substrates are either randomly or selectively taken up into endosomes. eMI substrates have KFERQ(-like) motifs and are delivered to endosomes by HSC70, reminiscent of CMA (^137^; see previous section) (Fig. 1d). However, eMI requires neither LAMP2A nor substrate unfolding^137^. As LAMP2A is found only in the genomes of mammals and birds, eMI might have emerged in other organisms to eliminate KFERQ-containing proteins^8^. Like multivesicular body formation and vacuolar microautophagy in yeast, membrane invagination in eMI is executed by the ESCRT machinery^137^ and partly HSC70^138^ which can deform membrane upon its binding to phosphatidylserine^137,139^ (Fig. 1d). After being incorporated into intraluminal vesicles, eMI substrates can be degraded within endosomes or lysosomes^137^. They can even be secreted out of the cell^140^. A similar process has also been found in fission yeast^141^.

Although the regulation of mammalian eMI is still mostly unknown, some hints can be derived from findings obtained from studies with *Drosophila*^8^. *Drosophila* eMI can be induced by starvation in a manner involving TOR (homologous to mTOR) inactivation^8^. Mammalian eMI may also be subjected to mTORC1-mediated regulation as it could be strongly induced by rapamycin treatment^9^. Like mammalian eMI, proteins of the ATG conjugation system are not involved in *Drosophila* eMI^8,137^. Further investigation revealed that ATG1 and ATG13, components of the macroautophagy initiation complex, are essential for *Drosophila* eMI^8^, which suggests that eMI and macroautophagy are regulated by the same upstream factors. Further indication of cross-talk between eMI and macroautophagy comes from the discovery that macroautophagy receptors are rapidly degraded by eMI during the first few hours of starvation in mammalian cells^142^.

eMI has been postulated to be the primary microautophagy pathway^8,137^, but the possibility of a lysosome-based microautophagy pathway still cannot be discounted. Although endosomes isolated from VPS4-depleted cells (and thus incapable of eMI) barely contain typical microautophagy substrates (cyclophilin, GAPDH and aldose), lysosomes from these cells have increased levels of the same proteins compared to those from normal cells^137^. On a related note, GAPDH puncta were still observed in cells depleted of both LAMP2A and TSG101 (a component of ESCRT-I)^143^ and could represent lysosomes. Although upregulation of CMA could explain the former observation and macroautophagy for the latter, they could also be due to lysosomes directly engulfing proteins for degradation as seen from earlier studies where isolated lysosomes were shown to be able to take up material such as Percoll particles^144^ and ferritin^144,145^.

There is still much to learn about microautophagy. Remaining questions include how it is regulated, what factors are involved, whether substrates are taken up specifically, whether membrane proteins are actively excluded (which has been demonstrated to occur for the V-ATPase in yeast microautophagy^146^ and indicated by the poor particle density of intravacuolar tubules^147^), and the extent of its physiological significance.

## Conclusion

Despite the fact that the lysosome is essential to autophagy, it has been mostly relegated to a role secondary to the autophagosome in studies on macroautophagy (the most well-characterized form of autophagy). Lysosomal function is intricately linked with that of autophagy: autophagic dysfunction is often caused by defective lysosomal activity as exemplified by the phenotypes of lysosomal storage diseases^148^. And yet, autophagy-related lysosomal defects are rarely characterized in detail. The extent of interdependency between autophagy machinery and lysosomal activation during starvation is also unclear. Even changes that occur to the lysosomal membrane and lumen during autophagy have only been partially described. Furthermore, microautophagy is a research field that is mostly unexplored. Increasing efforts to understand the lysosome is necessary to achieve a complete picture of autophagy.
